# Volatile Metabolites from *Brevundimonas diminuta* and Nematicidal Esters Inhibit *Meloidogyne javanica*

**DOI:** 10.3390/microorganisms11040966

**Published:** 2023-04-07

**Authors:** Yongxiang Sun, Yuan Ran, Hanbo Yang, Minghe Mo, Guohong Li

**Affiliations:** State Key Laboratory for Conservation and Utilization of Bio-Resources in Yunnan, School of Life Sciences, Yunnan University, Kunming 650091, China

**Keywords:** volatile metabolites, *Brevundimonas diminuta*, nematicidal activity, egg-hatching inhibitory, esters, *Meloidogyne javanica*

## Abstract

*Brevundimonas diminuta* is broadly distributed in terrestrial and aquatic environments and has various biological activities. In this study, we found that *B. diminuta* exhibited nematicidal activity against the plant root-knot nematode, *Meloidogyne javanica*. A total of 42 volatile organic compounds (VOCs) from *B. diminuta* were identified using gas chromatography–mass spectrometry (GC-MS). The nematicidal activity of the 10 main VOCs was tested against *M. javanica*. Butyl butanoate (4 µL) caused the mortality of 80.13% of *M. javanica* after 4 h. The nematicidal activity of an additional 38 butyl-butyrate-like volatile esters was also investigated. Of these, seven had strong nematicidal activity against *M. javanica*, five of which showed egg-hatching inhibitory activity. This study is the first to report that butyl butanoate, ethyl 2-methylbutanoate, ethyl 4-methylpentanoate, ethyl pent-4-enoate, and methyl undecanoate have nematicidal activity against *M. javanica*. The results indicated that *B. diminuta* could serve as a candidate microorganism for the biocontrol of plant root-knot nematodes, showing that volatile esters have great potential as nematicides.

## 1. Introduction

Plant parasitic nematodes are found in almost all crop types worldwide and are responsible for huge economic losses in the agricultural sector [1]. Of these, plant root-knot nematodes (*Meloidogyne* spp.), resident parasites of plant roots, are responsible for the most significant agricultural losses [2]. Chemical methods provide effective ways to control nematodes but are limited by their impact on the environment and human health. The biocontrol of plant parasitic nematodes has been increasingly studied because it increases agricultural output and has few negative environmental impacts [3]. Natural volatile organic compounds (VOCs) are essential to the biocontrol of plant parasitic nematodes and serve as an important potential resource for the development of nematicides [4].

Many volatile substances have a strong inhibitory effect on nematode survival, reproduction, development, and behavior, and have been studied as nematicidal, larvicidal, and repellent agents [5]. Microorganisms and plants provide abundant sources of natural volatile products against nematodes [6], many of which specifically inhibit plant root-knot nematodes. *Muscodor albus* secretes volatile mixtures for the control of root-knot nematodes in tomato roots [7]. *Eruca sativa* produces volatile metabolites such as erucin, pentyl isothiocyanate, hexyl isothiocyanate, (*E*)-2-hexenal, 2-ethylfuran, and methyl thiocyanate [8] that are active against *Meloidogyne incognita*. *Melia azedarach* also secretes volatile aldehydes and carboxylic acids, which are capable of killing *M. incognita* [9]. Furfural, (*E*,*E*)-2,4-decadienal, and (*E*)-2-decenal from *Ailanthus altissima* were shown to exhibit nematicidal activity against *M. javanica* [10]. Recently, many nematicidal volatile metabolites were also found in bacteria, such as *Pseudomonas* [11], *Comamonas* [11], *Variovorax* [11], *Bacillus* [12,13,14,15], *Lysinibacillus* [16], *Paenibacillus* [17], *Pseudochrobactrum* [18], *Proteus* [18], *Wautersiella* [18], *Arthrobacter* [18], *Achromobacter* [18], *Virgibacillus* [19,20], *Pseudoalteromonas* [21], and *Vibrio* [21]. 

*Brevundimonas diminuta* is a Gram-negative aerobic bacterium that was first isolated and identified in 1967 [22]. This species is abundant in various habitats and has diversiform bioactivities. *B. diminuta* isolated from arsenic-polluted soil can remove arsenic and promote plant growth [23], and *B. diminuta* obtained from mining soils can be used for the bioremediation of toxic metal contamination [24]. Studies have shown that *B. diminuta* can synthesize copper nanoparticles (CuNPs) with antibacterial activity [25]. *B. diminuta* can also degrade gentamicin and is an important candidate for the bioremediation of antibiotic contamination [26]. The *B. diminuta* strain isolated from the surface of iron ore and phosphorus ore can be used as a flotation agent to separate phosphorus and other harmful impurities [27]. *B. diminuta* can also degrade toxic substances in the textile dye rhodamine B [28] and aid the degradation of the pesticide chlorpyrifos [29]. Furthermore, these bacteria are used for the bioremediation of marine oil pollution [30]. They are resistant to silver nitrate environments and help to eliminate nitrates in groundwater [31]. As a rhizosphere bacterium, *B. diminuta* has the potential to serve as a cost-effective and environmentally friendly copper bioremediation agent [32], and as an ammonia-oxidizing bacterium, *B. diminuta* can remove organic ammonia [33]. *B. diminuta* is also used to produce nano-zinc and nano-zinc oxide [34], hydrolyze sulfide, degrade lubricating oil [35,36], and degrade foreign DNA into nucleic bases or their derivatives [37].

Few studies have evaluated the secondary metabolites of *B. diminuta*. A free lipid A produced by *Pseudomonas diminuta* (formerly named *Brevundimonas diminuta*) is able to function as an endotoxin [38]. The current study assessed the nematicidal activity of *B. diminuta* against the root-knot nematode *M. javanica* and measured its VOCs using GC-MS. The activity of the major VOCs against *M. javanica* was also assessed. Butyl butanoate was shown to exhibit strong activity against nematodes and egg-hatching inhibitory activity. The activity of 38 additional ester compounds that were structurally similar to butyl butanoate was also tested. Of these, seven compounds had high nematicidal activity, and four showed egg-hatching inhibitory activity. These findings provide a basis for the identification of active nematicides.

## 2. Materials and Methods

### 2.1. Experimental Strain and Culture

*Brevundimonas diminuta* (YMF3.00050) was preserved in the State Key Laboratory of Conservation and Utilization of Biological Resources in Yunnan, China. *Meloidogyne javanica* was obtained from the roots of tomatoes that were planted in the same laboratory.

The experimental strain was initially stored in nutrient broth (NB: 3.0 g beef extract, 10.0 g peptone, 5.0 g sodium chloride, 15.0 g agar, 1 L water, and pH nature) at 4 °C. The strain was transferred to an NB solid medium for 1 day at 28 °C and then cultivated in a sterilized 250 mL triangular flask with 100 mL of an NB liquid medium at 28 °C at 180 rpm for 2 days to develop the seed liquid. The seed solution (1 mL) was then transferred into a 500 mL sterile triangular flask filled with 200 mL of the NB liquid culture medium, cultured for 4 days at 28 °C, and shaken at 180 rpm. Part of the fermentation liquid was filtered through a 0.2 μm filter membrane to remove the bacteria, and the fermentation broth was collected in a 250 mL sterile vial.

The method of acquiring nematode eggs from roots was described previously [39]. In brief, *M. javanica* egg masses were handpicked from infected tomato roots, surface-sterilized in 0.5% NaClO solution for 3 min, rinsed 4–5 times with distilled water (dH_2_O) until the NaClO was removed, and incubated in a Petri dish with dH_2_O at 28 °C in the dark to prepare second-stage juveniles (J2s).

All the commercial compounds used in this study were purchased. Information on the manufacturer and purity of these compounds is provided in Tables S2 and S3.

### 2.2. Assay of Nematicidal Activity of Fermentation Broth against M. javanica

The *B. diminuta* fermentation broth (200 µL) was cultured in the NB medium in a well in the center of a 96-well plate. The nematode suspension (200 µL; approximately 100–150 worms) was added to eight wells surrounding the tested sample. The same amount of NB medium was used as a negative control. The 96-well plates were immediately sealed with a sealing film (Parafilm, Ocala, FL, USA) and incubated at 28 °C for 8, 24, 48, and 72 h. The number of active and dead nematodes was recorded under a microscope. The nematodes were considered dead if their bodies were stiff and remained inactive after stimulation with needles. The experiment was replicated three times.

### 2.3. Gas Chromatography–Mass Spectrometry Analysis

The fermentation broth of *B. diminuta* on the NB medium was extracted using cyclohexane to obtain volatile substances and dehydrated over anhydrous sodium sulfate, dried quickly using a rotary evaporator, and dissolved in 1 mL cyclohexane to prepare the sample. GC-MS (HP6890GC-5973MS GC-MS system, Agilent Technologies, Santa Clara, CA, USA) was used to analyze the VOC samples. For GC conditions, an HP-5 quartz capillary column (30 mm × 0.25 mm × 0.25 mm) was used. The initial column temperature was 40 °C, which was ramped at 3 °C/min to 80 °C and then ramped at 5 °C/min to 280 °C; this temperature was held for 10 min; the column flow rate was 1.0 mL/min; injection volume was 2.0 mL; the carrying gas was high-purity helium. For MS conditions, the ionization mode was EI.

In order to ensure that all the compounds were from *B. diminuta*, the NB medium was extracted to prepare the samples. According to the test results (Figure S1), the compounds detected in the NB medium samples were removed. The VOCs were identified according to a similarity index > 80%, by comparing the mass spectra (Figure S2) of the substance with the database (NIST) [40].

### 2.4. Nematicidal Activity of the Volatile Metabolites

The nematicidal activity of VOCs was tested according to the methods of Gu et al. [40] and Popova et al. [41], with some modifications. Different doses of commercial VOCs were added to a 3 cm Petri dish containing a WA medium (15% agar). A nematode suspension (5 µL) in ddH_2_O (about 150–200 worms) was then added to the center of the plate. Plates with no VOC samples were used as a blank control. The plates were immediately sealed with a sealing membrane (Parafilm, USA) and incubated at 28 °C for 1, 4, 8, and 24 h. The number of active and dead nematodes was recorded under a microscope. The experiment was repeated three times.

### 2.5. Ability of the Volatile Metabolites to Inhibit Egg Hatching

The inhibitory effect of VOCs on *M. javanica* egg hatching was assessed. The experiments were conducted in 96-well plates. Each VOC was individually added to a separate well in the center of a 96-well plate. Sterile water (200 µL) containing an egg mass was added to eight wells around the test sample, and the plates were incubated at 28 °C for 24, 48, and 72 h. The number of J2s was then counted under a microscope. The experiment was repeated three times.

### 2.6. Data Analysis and Statistics

The experimental data were analyzed using SPSS 20. The comparison between groups was analyzed through the single-factor ANOVA, and means were compared using the least significant differences (Duncan) at *p* = 0.05. LSD was used in the post hoc test.

## 3. Results

### 3.1. Nematicidal Activity of the B. diminuta Fermentation Broth against M. javanica

**Figure 1:**
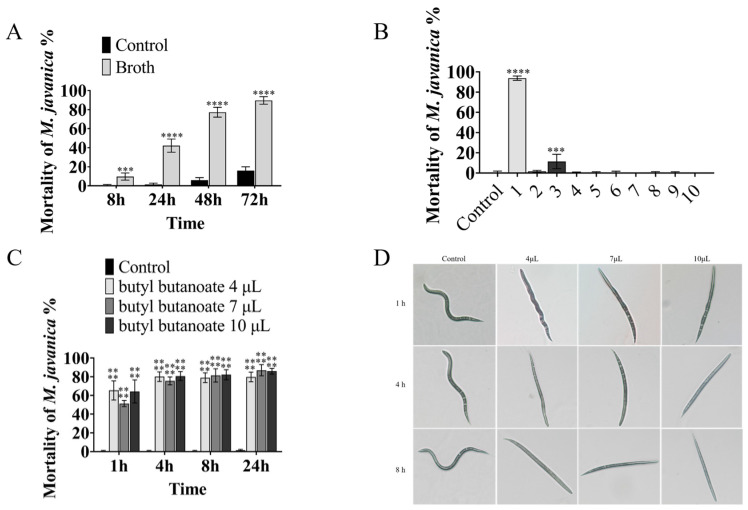
The nematicidal activity of the *B. diminuta*-specific VOCs. Bars refer to the standard error of the means (n = 3). The asterisk above the columns represents a significant difference using ANOVA, **** mean *p* < 0.0001; *** mean *p* < 0.001: (**A**) the mortality of nematodes after treatment with broth or control; (**B**) the nematicidal activity of 10 VOCs produced by *B. diminuta*, 1–10 were butyl butanoate, 2- (1-hydroxycyclohexyl)cyclohexan-1-one, 2- (cyclohexen-1-yl)cyclohexan-1-one, tetradecane, butylcyclohexane, dodecane, bis (6-methylheptyl) benzene-1,2-dicarboxylate, dibutyl benzene-1,2-dicarboxylate, (*6E*,*10E*,*14E*,*18E*)-2,6,10,15,19,23-hexamethyltetracosa-2,6,10,14,18,22-hexaene and cyclohexylcyclohexane; (**C**) different doses of butyl butanoate; (**D**) the status of butyl-butanoate-treated nematodes.

The nematicidal activity of VOCs from the *B. diminuta* fermentation broth was determined in 96-well plates. The broth had strong activity against *M. javanica*, causing 89.67% mortality at 72 h, as compared with 16.11% mortality in the control group (Figure 1A).

### 3.2. Identification of VOCs Produced by B. diminuta

A total of 42 VOCs were identified in the *B. diminuta* fermentation broth extract (Table S1). The 10 most significant metabolites (Table 1) were selected to assay nematicidal activity according to their peak areas, quality, and structure. The nematicidal activity of each metabolite was tested using commercially available compounds (Table S2).

### 3.3. Nematicidal Activity of the VOCs Produced by B. diminuta

**Figure 2:**
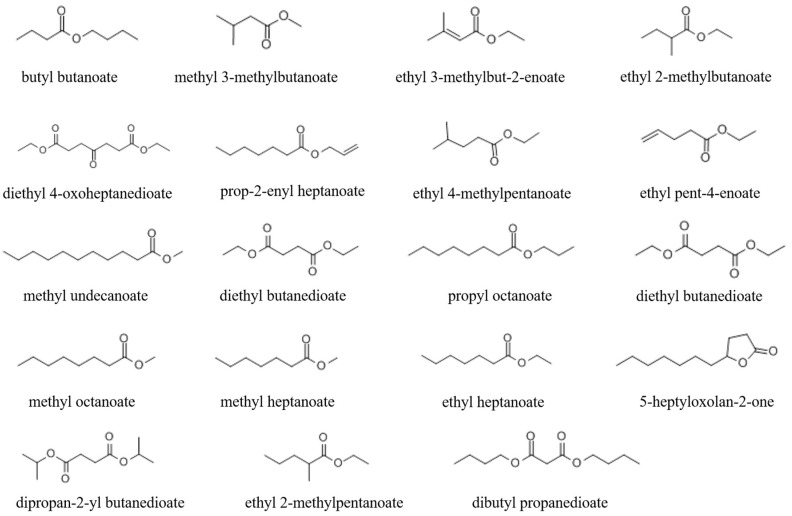
The structure of nematicidal volatile esters.

The nematicidal activity of VOCs was determined using a 3 cm Petri dish containing the WA medium (15% agar). While butyl butanoate (Figure 2) (10 µL) showed strong nematicidal activity against *M. javanica*, causing the mortality of 93.75% of *M. javanica* in 24 h, the other nine compounds had no evident activity (Table 2 and Figure 1B). The activity of butyl butanoate against *M. javanica* was also shown at lower doses. At 4 µL, butyl butanoate caused the mortality of 80.13% of *M. javanica* at 4 h, as compared with 0.56% mortality in the control group (Figure 1C). After butyl butanoate treatment, the nematodes became sluggish, gradually lost mobility at 1 h, and then became stiff and died (Figure 1D).

### 3.4. Nematicidal Activity of the Volatile Ester Compounds

**Figure 3:**
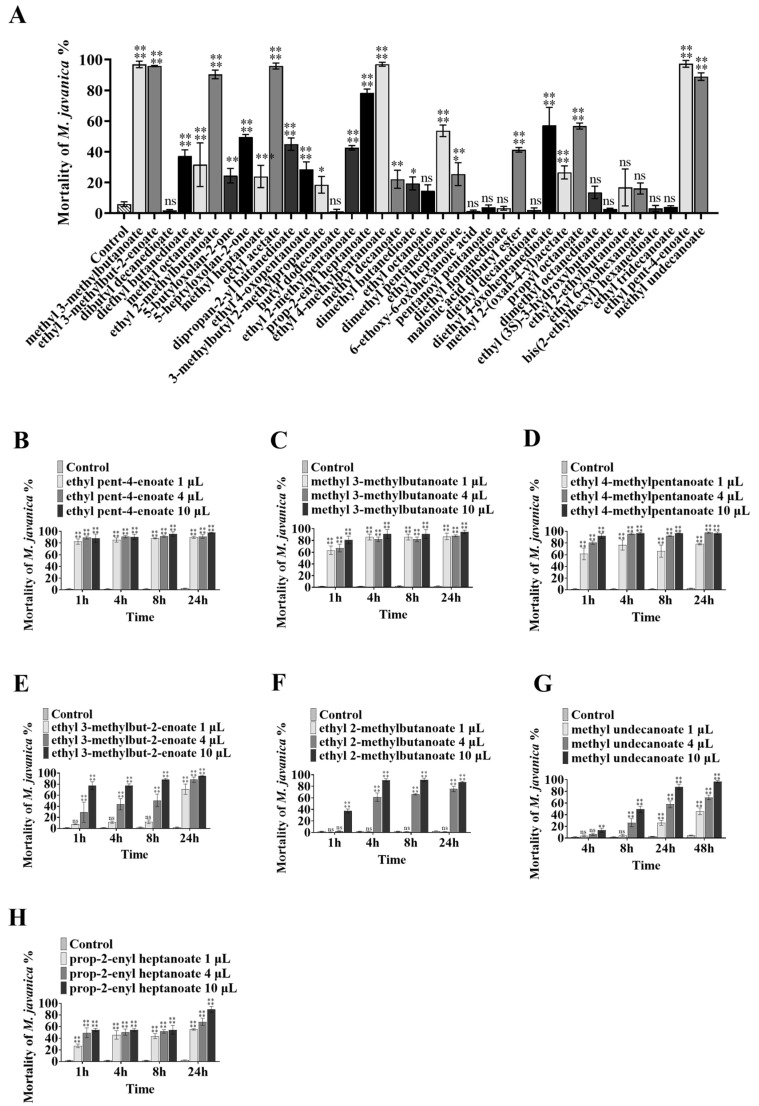
The mortality of nematodes treated with different VOCs at different doses. Bars represent the standard error of the means (n = 3). The asterisk symbols above the columns signify significant differences using ANOVA, **** mean *p* < 0.0001; *** mean *p* < 0.001; ** mean *p* < 0.01; * mean *p* < 0.05: (**A**) treatment with different VOCs; treatment with different doses of (**B**) ethyl pent-4-enoate, (**C**) methyl 3-methylbutanoate, (**D**) ethyl 4-methylpentanoate, (**E**) ethyl 3-methylbut-2-enoate, (**F**) ethyl 2-methylbutanoate, (**G**) methyl undecanoate, and (**H**) prop-2-enyl heptanoate.

To further explore the active substances of volatile esters, 38 compounds with structures like butyl butanoate (Table S3) were obtained to investigate their nematicidal activity. At a dose of 10 µL, 7 of the 38 compounds (methyl 3-methylbutanoate, ethyl 3-methylbut-2-enoate, ethyl 2-methylbutanoate, prop-2-enyl heptanoate, ethyl 4-methylpentanoate, ethyl pent-4-enoate, and methyl undecanoate) exhibited strong activity against *M. javanica*. At 24 h, these compounds caused the mortality of 94.66%, 94.75%, 85.98%, 89.7%, 89.69%, 90.85%, and 87.85% of *M. javanica*, respectively (Figure 3A). At 72 h, seven compounds (diethyl butanedioate, 5-heptyloxolan-2-one, dipropan-2-yl butanedioate, ethyl 2-methylpentanoate, dibutyl propanedioate, diethyl 4-oxoheptanedioate, and propyl octanoate) had a weak nematicidal effect, causing 37.35%, 49.72%, 44.98%, 42.61%, 41.28%, 57.29%, and 56.74% mortality of *M. javanica*, respectively (Figure 3A). Four metabolites (diethyl butanedioate, methyl octanoate, propyl octanoate, and ethyl heptanoate) inhibited nematode mobility but did not cause them to become stiff and die. The structures of these active compounds are shown in Figure 2.

The nematicidal ability of the seven compounds with high activities was further assayed at lower doses. Ethyl pent-4-enoate and methyl 3-methylbutanoate were both shown to have strong nematicidal activity, while 1 µL ethyl pent-4-enoate caused 82.38% mortality of *M. javanica* at 1 h (Figure 3B), and 1 µL methyl 3-methylbutanoate caused 85.82% mortality after 8 h (Figure 3C). At 4 µL, ethyl 4-methylpentanoate caused 95.69% mortality after 4 h (Figure 3D), and ethyl 3-methylbut-2-enoate, ethyl 2-methylbutanoate, methyl undecanoate, and prop-2-enyl heptanoate caused 88.08% (Figure 3E), 75.51% (Figure 3F), 57.83% (Figure 3G), and 68.29% (Figure 3H) mortality after 24 h, respectively.

### 3.5. Inhibition of Egg-Hatching Activity by Volatile Ester Compounds

**Figure 4:**
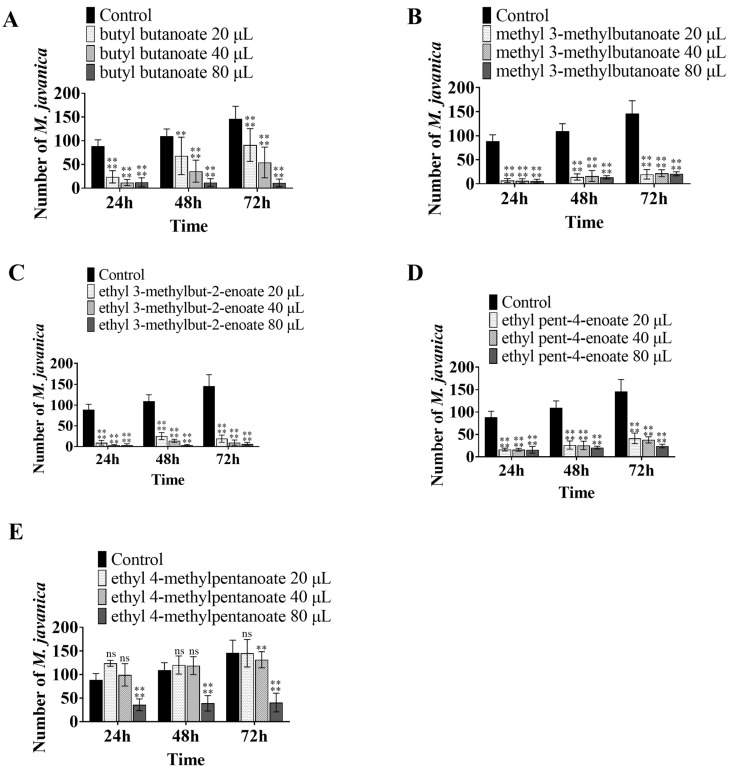
Inhibition of egg-hatching activity by VOCs. Bars represent the standard error of the means (n = 3). The asterisk above the columns indicates significant differences using ANOVA, **** mean *p* < 0.0001; ** mean *p* < 0.01: (**A**) the number of nematodes hatched in response to treatment with different doses of (**A**) butyl butanoate, (**B**) methyl 3-methylbutanoate, (**C**) ethyl 3-methylbut-2-enoate, (**D**) ethyl pent-4-enoate, and (**E**) ethyl 4-methylpentanoate.

The seven VOCs, i.e., butyl butanoate, methyl 3-methylbutanoate, ethyl 3-methylbut-2-enoate, ethyl 2-methylbutanoate, ethyl 4-methylpentanoate, ethyl pent-4-enoate, and methyl undecanoate, were tested for their ability to inhibit egg hatching. Butyl butanoate, methyl 3-methylbutanoate, ethyl 3-methylbut-2-enoate, and ethyl pent-4-enoate showed high inhibitory activity. After treatment with 40 μL butyl butanoate, 20 μL methyl 3-methylbutanoate, ethyl 3-methylbut-2-enoate, and ethyl pent-4-enoate, the average number of hatching worms per egg mass were 56.17, 19.67, 19.0, and 41.17, respectively, after 72 h (Figure 4A–D), while the number of hatching worms in the control group was 145.89. Ethyl 4-methylpentanoate had a weak inhibitory effect on egg hatching. After treatment with 80 μL ethyl 4-methylpentanoate, the average number of hatching worms from each egg mass was 40.5 after 72 h (Figure 4E). Ethyl 2-methylbutanoate and methyl undecanoate had no obvious inhibitory activity. 

## 4. Discussion

Root-knot nematodes are usually infected by bacteria and fungi, which also makes bacteria potential resources for controlling nematodes [42]. Several studies have indicated that bacteria and bacterial metabolites have great potential for use as biocontrol agents [43,44,45]. The current study was the first to show that *B. diminuta* had nematicidal activity against *M. javanica* by producing volatile substances, providing a valuable microbial resource for the biological control of nematodes.

VOCs have a small molecular weight, a high vapor pressure, and a low boiling point [46], and can kill insects or inhibit bacteria via fumigation at a certain temperature. Studies have assessed the nematicide activity of VOCs produced by various bacteria [40]. Four VOCs produced by deep-sea bacteria have been shown to have nematicidal and attractive activities against *M. incognita* [20]. Methyl thioacetate produced by bacteria has been found to control *M. incognita* by contacting and fumigating nematodes [47]. Cheng et al. showed that volatile compounds from bacteria can resist *Meloidogyne* in a number of ways, and 2-nonanone and 2-decanone can kill nematodes by damaging the nematode gut [17]. Some bacteria collected from the soil where nematodes exist can produce a variety of nematicides, including sulfuric, alkenes, and pyrazine [11]. Comparatively, natural products have more safety than chemicals, but whether they are truly safe requires further confirmation.

*B. diminuta* has diversiform bioactivities, but its role in the control of plant parasitic nematodes has remained unknown. Thus, this study sought to identify nematicidal VOCs from *B. diminuta*. The VOCs produced by *B. diminuta* were analyzed using GC-MS, and the nematicidal activity of the major VOCs was determined. Butyl butanoate was shown to have strong nematicidal activity. At a dose of 4 μL, nematode mortality was 80.13% after 4 h. To further characterize VOCs with nematicidal activity, the activity of 38 compounds with structures like butyl butanoate was tested. Seven compounds had promising activity against *M. incognita*. In addition, five metabolites (butyl butanoate, methyl 3-methylbutanoate, ethyl 3-methylbut-2-enoate, ethyl 4-methylpentanoate, and ethyl pent-4-enoate) showed significant inhibitory activity on *M. javanica* egg hatching. This is the first study to report on the nematicidal activity of butyl butanoate, ethyl 2-methylbutanoate, ethyl 4-methylpentanoate, ethyl pent-4-enoate, and methyl undecanoate. The nematicidal activity of methyl 3-methylbutanoate and the insecticidal activity of ethyl 3-methylbut-2-enoate have been demonstrated previously [18]. While prop-2-enyl heptanoate was shown to be toxic for insect cells [48], no report assessed its effect on nematodes. The results of the current study are consistent with those of previous studies, indicating that volatile ester compounds have great potential for nematode control.

Several studies have assessed the nematicidal activity of esters. For example, ethyl caproate has nematicidal activity against *M. incognita* [49]. Zhai et al. found that (Z)-hexen-1-ol acetate produced by *Pseudomonas putida* has extremely strong nematicidal activity [50]. Butyl butanoate, methyl 3-methylbutanoate, ethyl 3-methylbut-2-enoate, ethyl 4-methylpentanoate, and ethyl pent-4-enoate were shown to control *M. javanica* by directly killing and inhibiting egg hatching. Compared with traditional nematicides with a single activity, these compounds have greater advantages for the development of insecticides.

## 5. Conclusions

This study found that *B. diminuta* fermentation broth was active against nematodes. Notably, 42 VOCs were identified via GC-MS analysis, among which butyl butanoate had both nematicidal and egg-hatching inhibition activity. This strain can serve as a valuable microbial resource for the biological control of nematodes. Testing the activity of 38 ester VOCs with a similar structure to butyl butanoate revealed that methyl 3-methylbutanoate, ethyl 3-methylbut-2-enoate, ethyl 4-methylpentanoate, and ethyl pent-4-enoate also had significant nematicidal and egg-hatching inhibition activity, while ethyl 2-methylbutanoate, methyl undecanoate, and allyl heptanoate had significant nematicidal activity. These findings indicate that ester volatile compounds have great potential for the development of nematode control agents. However, in practical production and application, how to develop nematicides of volatile substances that can give full play to nematodes needs further in-depth study.

## Figures and Tables

**Table 1:** The major 10 VOCs produced by *B. diminuta*.

Components	CAS No.	Formula	Molecular Weight	RT (min)	Area %	Qual %
butyl butanoate	109-21-7	C_8_H_16_O_2_	144	10.61	0.45	91
2-(1-hydroxycyclohexyl)cyclohexan-1-one	28746-99-8	C_12_H_20_O_2_	196	30.76	0.88	86
2-(cyclohexen-1-yl)cyclohexan-1-one	1502-22-3	C_12_H_18_O	178	28.73	0.55	83
tetradecane	629-59-4	C_14_H_30_	198	25.31	0.84	98
butylcyclohexane	1678-93-9	C_10_H_20_	140	11.97	0.35	97
dodecane	112-40-3	C_12_H_26_	170	19.00	0.3	94
bis(6-methylheptyl) benzene-1,2-dicarboxylate	27554-26-3	C_24_H_38_O_4_	391	49.52	6.99	91
dibutyl benzene-1,2-dicarboxylate	84-74-2	C_16_H_22_O_4_	278	38.77	0.56	96
(*6E*,*10E*,*14E*,*18E*)-2,6,10,15,19,23-hexamethyltetracosa-2,6,10,14,18,22-hexaene	111-02-4	C_30_H_50_	411	53.68	0.31	98
cyclohexylcyclohexane	92-51-3	C_12_H_22_	166	22.33	0.67	96

**Table 2:** The mortality of *M. javanica* treated with 10 VOCs (10 µL) produced by *B. diminuta*.

Compounds	Mortality of *M. javanica* (%) ± SD
	24 h
butyl butanoate	93.75 ± 0.02
2-(1-hydroxycyclohexyl)cyclohexan-1-one	1.80 ± 0.01
2-(cyclohexen-1-yl)cyclohexan-1-one	11.39 ± 0.08
tetradecane	0.37 ± 0.01
butylcyclohexane	0.71 ± 0.01
dodecane	0.65 ± 0.01
bis(6-methylheptyl) benzene-1,2-dicarboxylate	0.37 ± 0.01
dibutyl benzene-1,2-dicarboxylate	2.24 ± 0.01
(*6E*,*10E*,*14E*,*18E*)-2,6,10,15,19,23-hexamethyltetracosa-2,6,10,14,18,22-hexaene	0.70 ± 0.00
cyclohexylcyclohexane	0 ± 0.00
control	0.71 ± 0.01

## Data Availability

Not applicable.

## Supplementary Materials

The following supporting information can be downloaded at: https://www.mdpi.com/article/10.3390/microorganisms11040966/s1, Supplementary File: Supplementary information; Table S1: Forty-two volatile organic compounds identified from *B. diminuta* via GC-MS; Table S2: Commercial information of 10 VOCs used to test nematicidal activity; Table S3: Thirty-eight butyl-butyrate-like volatile esters and commercial information; Figure S1: The GC-MS chromatogram of NB medium; Figure S2: The GC-MS chromatogram of *B. diminuta*.

## References

[1] Elling A.A. (2013). Major Emerging Problems with Minor *Meloidogyne* Species. Phytopathology.

[2] Jones J.T., Haegeman A., Danchin E.G.J., Gaur H.S., Helder J., Jones M.G.K., Kikuchi T., Manzanilla-López R., Palomares-Rius J.E., Wesemael W.M.L. (2013). Top 10 plant-parasitic nematodes in molecular plant pathology. Mol. Plant Pathol..

[3] Tapia-Vázquez I., Montoya-Martínez A.C., De los Santos-Villalobos S., Ek-Ramos M.J., Montesinos-Matías R., Martínez-Anaya C. (2022). Root-knot nematodes (*Meloidogyne* spp.) a threat to agriculture in Mexico: Biology, current control strategies, and perspectives. World J. Microbiol. Biotechnol..

[4] Andrés M.F., González-Coloma A., Sanz J., Burillo J., Sainz P. (2012). Nematicidal activity of essential oils: A review. Phytochem. Rev..

[5] Piao X., Sun M., Yi F. (2020). Evaluation of Nematocidal Action against *Caenorhabditis elegans* of Essential Oil of Flesh Fingered Citron and Its Mechanism. J. Chem..

[6] Deng X., Wang X., Li G. (2022). Nematicidal Effects of Volatile Organic Compounds from Microorganisms and Plants on Plant-Parasitic Nematodes. Microorganisms.

[7] Grimme E., Zidack N.K., Sikora R.A., Strobel G.A., Jacobsen B.J. (2007). Comparison of Muscodor albus Volatiles with a Biorational Mixture for Control of Seedling Diseases of Sugar Beet and Root-Knot Nematode on Tomato. Plant Dis..

[8] Aissani N., Urgeghe P.P., Oplos C., Saba M., Tocco G., Petretto G.L., Eloh K., Menkissoglu-Spiroudi U., Ntalli N., Caboni P. (2015). Nematicidal Activity of the Volatilome of Eruca sativa on *Meloidogyne incognita*. J. Agric. Food Chem..

[9] Ntalli N.G., Vargiu S., Menkissoglu-Spiroudi U., Caboni P. (2010). Nematicidal Carboxylic Acids and Aldehydes from *Melia azedarach* Fruits. J. Agric. Food Chem..

[10] Caboni P., Ntalli N.G., Aissani N., Cavoski I., Angioni A. (2012). Nematicidal Activity of (*E*,*E*)-2,4-Decadienal and (*E*)-2-Decenal from *Ailanthus altissima* against *Meloidogyne javanica*. J. Agric. Food Chem..

[11] Wolfgang A., Taffner J., Guimarães R.A., Coyne D., Berg G. (2019). Novel Strategies for Soil-Borne Diseases: Exploiting the Microbiome and Volatile-Based Mechanisms Toward Controlling *Meloidogyne* Based Disease Complexes. Front. Microbiol..

[12] Ayaz M., Ali Q., Farzand A., Khan A.R., Ling H., Gao X. (2021). Nematicidal Volatiles from *Bacillus atrophaeus* GBSC56 Promote Growth and Stimulate Induced Systemic Resistance in Tomato against *Meloidogyne incognita*. Int. J. Mol. Sci..

[13] Huang Y., Xu C., Ma L., Zhang K., Duan C., Mo M. (2010). Characterisation of volatiles produced from *Bacillus megaterium* YFM3.25 and their nematicidal activity against *Meloidogyne incognita*. Eur. J. Plant Pathol..

[14] Luo T., Hou S., Yang L., Qi G., Zhao X. (2018). Nematodes avoid and are killed by *Bacillus mycoides* produced styrene. J. Invertebr. Pathol..

[15] Yin N., Liu R., Zhao J.L., Khan R.A.A., Li Y., Ling J., Liu W., Yang Y.H., Xie B.Y., Mao Z.C. (2021). Volatile Organic Compounds of *Bacillus cereus* Strain Bc-cm103 Exhibit Fumigation Activity against *Meloidogyne incognita*. Plant Dis..

[16] Yang L.-L., Huang Y., Liu J., Ma L., Mo M.-H., Li W.-J., Yang F.-X. (2012). *Lysinibacillus mangiferahumi* sp. nov., a new bacterium producing nematicidal volatiles. Antonie Leeuwenhoek.

[17] Cheng W., Yang J., Nie Q., Huang D., Yu C., Zheng L., Cai M., Thomashow L.S., Weller D.M., Yu Z. (2017). Volatile organic compounds from *Paenibacillus polymyxa* KM2501-1 control *Meloidogyne incognita* by multiple strategies. Sci. Rep..

[18] Xu Y.-Y., Lu H., Wang X., Zhang K.-Q., Li G.-H. (2015). Effect of Volatile Organic Compounds from Bacteria on Nematodes. Chem. Biodiversity.

[19] Huang D., Hao Y.U., Wen C., Wanli C., Zongze S., Jibin Z. (2022). Identification and characteristics of 4-vinylphenol from *Virgibacillus dokdonensis* MCCC 1A00493 against *Meloidogyne incognita*. Acta Microbiol. Sin..

[20] Huang D., Yu C., Shao Z., Cai M., Li G., Zheng L., Yu Z., Zhang J. (2020). Identification and Characterization of Nematicidal Volatile Organic Compounds from Deep-Sea *Virgibacillus dokdonensis* MCCC 1A00493. Molecules.

[21] Yu J., Du G.C., Li R.G., Li L., Li Z., Zhou C.J., Chen C.C., Guo D.S. (2015). Nematicidal activities of bacterial volatiles and components from two marine bacteria, *Pseudoalteromonas marina* strain H-42 and *Vibrio atlanticus* strain S-16, against the pine wood nematode, *Bursaphelenchus xylophilus*. Nematology.

[22] Bowman F.W., Calhoun M.P., White M. (1967). Microbiological Methods for Quality Control of Membrane Filters. J. Pharm. Sci..

[23] Singh N., Marwa N., Mishra S.K., Mishra J., Verma P.C., Rathaur S., Singh N. (2016). *Brevundimonas diminuta* mediated alleviation of arsenic toxicity and plant growth promotion in *Oryza sativa* L.. Ecotoxicol. Environ. Saf..

[24] Ali A., Li M., Su J., Li Y., Wang Z., Bai Y., Ali E.F., Shaheen S.M. (2022). *Brevundimonas diminuta* isolated from mines polluted soil immobilized cadmium (Cd^2+^) and zinc (Zn^2+^) through calcium carbonate precipitation: Microscopic and spectroscopic investigations. Sci. Total Environ..

[25] Joshi M.H., Patil A.A., Chaudhary S., Kale R.D. (2020). Microbial synthesis of CuNPs using *Brevundimonas diminuta* strain and its antibacterial activity. Adv. Nat. Sci. Nanosci. Nanotechnol..

[26] Liu Y., Chang H., Li Z., Feng Y., Cheng D., Xue J. (2017). Biodegradation of gentamicin by bacterial consortia AMQD4 in synthetic medium and raw gentamicin sewage. Sci. Rep..

[27] Abdel-Khalek N., Selim K., Abdallah S., Yassin K. (2013). Bioflotation of low Grade Egyptian Iron ore using *Brevundimonas diminuta* Bacteria: Phosphorus removal. Elixir Bio Technol..

[28] Saravanan S., Carolin C.F., Kumar P.S., Chitra B., Rangasamy G. (2022). Biodegradation of textile dye Rhodamine-B by *Brevundimonas diminuta* and screening of their breakdown metabolites. Chemosphere.

[29] Hossain M.S., Chowdhury M.A.Z., Pramanik M.K., Rahman M.A., Fakhruddin A.N.M., Alam M.K. (2014). Determination of selected pesticides in water samples adjacent to agricultural fields and removal of organophosphorus insecticide chlorpyrifos using soil bacterial isolates. Appl. Water Sci..

[30] Wang X., Wang X., Liu M., Zhou L., Gu Z., Zhao J. (2015). Bioremediation of marine oil pollution by *Brevundimonas diminuta*: Effect of salinity and nutrients. Desalin. Water Treat..

[31] Kavitha S., Selvakumar R., Sathishkumar M., Swaminathan K., Lakshmanaperumalsamy P., Singh A., Jain S.K. (2009). Nitrate removal using *Brevundimonas diminuta* MTCC 8486 from ground water. Water Sci. Technol..

[32] Rathi M., Yogalakshmi K.N. (2021). Brevundimonas diminuta MYS6 associated Helianthus annuus L. for enhanced copper phytoremediation. Chemosphere.

[33] Kwon H.-K., Jung J.-O. (2007). Isolation and Characteristics of Novel Ammonia Oxidizing Bacteria *Brevundimonas diminuta*. Korean J. Environ. Health Sci..

[34] Mirhendi M., Emtiazi G., Roghanian R. (2013). Production of nano zinc, zinc sulphide and nanocomplex of magnetite zinc oxide by *Brevundimonas diminuta* and *Pseudomonas stutzeri*. IET Nanobiotechnol..

[35] Basuki W. (2017). Biodegradation of Used Synthetic Lubricating Oil by *Brevundimonas diminuta* AKL 1.6. Makara J. Sci..

[36] Oh M.-H., Lee E.-Y. (2017). Reduction of Sulfur Compounds Produced from Swine Manure, Using *Brevundimonas diminuta*. Microbiol. Biotechnol. Lett..

[37] Kruszewska H., Misicka A., Chmielowiec U. (2004). Biodegradation of DNA and nucleotides to nucleosides and free bases. Farmaco.

[38] Kasai N., Arata S., Mashimo J.-i., Akiyama Y., Tanaka C., Egawa K., Tanaka S. (1987). *Pseudomonas diminuta* LPS with a new endotoxic lipid A structure. Biochem. Biophys. Res. Commun..

[39] Liu R., Bao Z.-X., Li G.-H., Li C.-Q., Wang S.-L., Pan X.-R., Zhang K.-Q., Zhao P. (2022). Identification of Nematicidal Metabolites from *Purpureocillium lavendulum*. Microorganisms.

[40] Gu Y.-Q., Mo M.-H., Zhou J.-P., Zou C.-S., Zhang K.-Q. (2007). Evaluation and identification of potential organic nematicidal volatiles from soil bacteria. Soil Biol. Biochem..

[41] Popova A.A., Koksharova O.A., Lipasova V.A., Zaitseva J.V., Katkova-Zhukotskaya O.A., Eremina S.I., Mironov A.S., Chernin L.S., Khmel I.A. (2014). Inhibitory and Toxic Effects of Volatiles Emitted by Strains of *Pseudomonas* and *Serratia* on Growth and Survival of Selected Microorganisms, *Caenorhabditis elegans* and *Drosophila melanogaster*. BioMed. Res. Int..

[42] Zhang X., Guan P., Wang Y., Li Q., Zhang S., Zhang Z., Bezemer T.m., Liang W. (2015). Community composition, diversity and metabolic footprints of soil nematodes in differently-aged temperate forests. Soil Biol. Biochem..

[43] Arthurs S., Dara S.K. (2019). Microbial biopesticides for invertebrate pests and their markets in the United States. J. Invertebr..

[44] Ma Y.-y., Li Y.-l., Lai H.-x., Guo Q., Xue Q.-h. (2017). Effects of two strains of Streptomyces on root-zone microbes and nematodes for biocontrol of root-knot nematode disease in tomato. Appl. Soil Ecol..

[45] Zhai Y., Shao Z., Cai M., Zheng L., Li G., Yu Z., Zhang J. (2019). Cyclo(l-Pro–l-Leu) of *Pseudomonas putida* MCCC 1A00316 Isolated from Antarctic Soil: Identification and Characterization of Activity against *Meloidogyne incognita*. Molecules.

[46] Pagans E., Font X., Sanchez A. (2006). Emission of volatile organic compounds from composting of different solid wastes: Abatement by biofiltration. J. Hazard. Mater..

[47] Chen W., Wang J., Huang D., Cheng W., Shao Z., Cai M., Zheng L., Ziniu y., Zhang J. (2021). Volatile Organic Compounds from *Bacillus aryabhattai* MCCC 1K02966 with Multiple Modes against *Meloidogyne incognita*. Molecules.

[48] Giner M., Avilla J., Balcells M., Caccia S., Smagghe G. (2012). Toxicity of allyl esters in insect cell lines and in Spodoptera littoralis larvae. Arch. Insect Biochem. Physiol..

[49] Silva M., Campos V., Terra W., Magalhães Pacheco P., Paula L., Barros A., Pedroso M. (2021). Volatile fatty acids from whey volatilome as potential soil fumigants to control *Meloidogyne incognita*. Crop Prot..

[50] Zhai Y., Shao Z., Cai M., Zheng L., Li G., Huang D., Cheng W., Thomashow L.S., Weller D.M., Yu Z. (2018). Multiple Modes of Nematode Control by Volatiles of *Pseudomonas putida* 1A00316 from Antarctic Soil against *Meloidogyne incognita*. Front. Microbiol..

