# Supplementary material for: Volatile Metabolites from Brevundimonas diminuta and Nematicidal Esters Inhibit Meloidogyne javanica
Source: Microorganisms. 2023 Apr 7;11(4):966. doi: 10.3390/microorganisms11040966 (PMC10144101; doi:10.3390/microorganisms11040966)
Supplement: Supplementary file 1 [file microorganisms-11-00966-s001.zip › microorganisms-2280993-supplementary.pdf]

Supplementary materials

Volatile Metabolites from *Brevundimonas diminuta* and Nematicidal Esters Inhibit *Meloidogyne javanica*

Yongxiang Sun, Yuan Ran, Hanbo Yang, Minghe Mo * and Guohong Li *

State key Laboratory for Conservation and Utilization of Bio-Resources in Yunnan, School of Life Sciences, Yunnan University, Kunming, Yunnan 650091, China

* Correspondence: ligh@ynu.edu.cn (G.L.); minghemo@163.com (M.M.)

Content

1. Table S1

2. Table S2

3. Table S3

4. Figure S1

5. Figure S2

Table S1. Forty-two volatile organic compounds identified from *B. diminuta* via GC-MS.

No.	Compounds	CAS No.	RT(min)	Area %	Qual %
1	hexanal	66-25-1	4.04	0.30	90
2	N,N-dimethylcyclohexanamine	98-94-2	9.78	0.50	91
3	butyl butanoate	109-21-7	10.61	0.45	91
4	butylcyclohexane	1678-93-9	11.97	0.35	97
5	nonanal	124-19-6	15.35	0.22	96
6	butoxycyclohexane	24072-44-4	15.67	0.43	94
7	1-phenylpropan-2-one	103-79-7	16.28	0.31	94
8	2-propylhept-2-enal	34880-43-8	18.45	0.25	92
9	dodecane	112-40-3	19.00	0.30	94
10	cyclohexylcyclohexane	92-51-3	22.33	0.67	96
11	tetradecane	629-59-4	25.31	0.84	98
12	cyclohexyl hexanoate	6243-10-3	26.03	0.24	80
13	2,6-ditert-butylcyclohexa-2,5-diene-1,4-dione	719-22-2	27.24	0.46	90
14	cyclohexylidenecyclohexane	4233-18-5	28.42	0.39	83
15	2-(cyclohexen-1-yl)cyclohexan-1-one	1502-22-3	28.73	0.55	83
16	3-methylpentadecane	2882-96-4	29.86	0.32	90
17	2-(1-hydroxycyclohexyl)cyclohexan-1-one	28746-99-8	30.76	0.88	86
18	heptadecane	629-78-7	32.96	0.30	97
19	2,6,10,14-tetramethylpentadecane	1921-70-6	33.10	0.24	98
20	dibutyl benzene-1,2-dicarboxylate	84-74-2	38.77	0.56	96
21	N-phenylnaphthalen-1-amine	90-30-2	44.08	0.22	87
22	(2,5-dimethoxy-4-propan-2-ylphenyl)methanol	62458-52-0	47.47	0.28	80
23	bis(6-methylheptyl) benzene-1,2-dicarboxylate	27554-26-3	49.52	6.99	91
24	9-methylnonadecane	13287-24-6	51.71	0.53	95
25	(6E,10E,14E,18E)-2,6,10,15,19,23-hexamethyltetracosa-2,6,10,14,18,22-hexaene	111-02-4	53.68	0.31	98
26	nonacosane	630-03-5	54.77	0.59	99
27	hexadecane	544-76-3	30.59	0.71	96
28	octadecane	593-45-3	35.24	0.39	94
29	undecylcyclopentane	6785-23-5	36.59	0.33	90
30	hexadecanoic acid	57-10-3	39.11	2.34	99
31	icosane	112-95-8	39.46	0.29	99
32	henicosane	629-94-7	41.44	0.38	99
33	hexadecanamide	629-54-9	43.13	0.40	94
34	docosane	629-97-0	43.32	0.40	99
35	(Z)-octadec-9-enamide	301-02-0	46.38	1.32	99
36	tetracosane	646-31-1	46.87	0.52	99
37	hexacosane	630-01-3	50.17	0.54	99
38	(Z)-docos-13-enamide	112-84-5	53.03	1.31	99
39	nonadecane	629-92-5	56.58	0.73	96

40	pentacosane	629-99-2	58.72	0.80	97
41	octacosane	630-02-4	61.32	0.68	99
42	triacontane	638-68-6	68.34	0.55	97

Table S2. Commercial information of 10 VOCs used to test nematicidal activity.

No.	Components	CAS No.	Product code	Purity	Brand
1	butyl butanoate	109-21-7	B802161	99.0 %	Macklin
2	2-(1-hydroxycyclohexyl)cyclohexan-1-one	28746-99-8	H908848	95.0 %	Macklin
3	2-(cyclohexen-1-yl)cyclohexan-1-one	1502-22-3	C806024	85.0 %	Macklin
4	tetradecane	629-59-4	T818478	98.0 %	Macklin
5	butylcyclohexane	1678-93-9	B871393	99.0 %	Macklin
6	dodecane	112-40-3	D807186	98.0 %	Macklin
7	bis(6-methylheptyl) benzene-1,2-dicarboxylate	27554-26-3	D822209	99.0 %	Macklin
8	dibutyl benzene-1,2-dicarboxylate	84-74-2	D806672	98.5 %	Macklin
9	(6E,10E,14E,18E)-2,6,10,15,19,23-hexamethyl tetracosa-2,6,10,14,18,22-hexaene	111-02-4	S817864	98.0 %	Macklin
10	cyclohexylcyclohexane	92-51-3	B802413	99.0 %	Macklin

Table S3. Thirty-eight butyl-butyrate-like volatile esters and commercial information.

No.	Compounds	CAS No.	Formula	Product code	Purity	Brand
1	methyl 3-methylbutanoate	556-24-1	$C_6H_{12}O_2$	65356A	98 %	Adamas
2	dimethyl octanedioate	1732-09-8	$C_{10}H_{18}O_4$	D840213	98 %	Macklin
3	ethyl 4-methylpentanoate	25415-67-2	$C_8H_{16}O_2$	E915660	97 %	Macklin
4	ethyl 3-methylbut-2-enoate	638-10-8	$C_7H_{12}O_2$	E835450	98 %	Macklin
5	dibutyl propanedioate	1190-39-2	$C_{11}H_{20}O_4$	D862583	99 %	Macklin
6	pentanoyl pentanoate	2082-59-9	$C_{10}H_{18}O_3$	V831004	98 %	Macklin
7	dipropan-2-yl butanedioate	924-88-9	$C_{10}H_{18}O_4$	D830992	98 %	Macklin
8	diethyl nonanedioate	624-17-9	$C_{13}H_{24}O_4$	D875497	90 %	Macklin
9	propyl octanoate	624-13-5	$C_{11}H_{22}O_2$	P860117	98 %	Macklin
10	ethyl tridecanoate	28267-29-0	$C_{15}H_{30}O_2$	E863573	97 %	Macklin
11	ethyl 2-ethylbutanoate	2983-38-2	$C_8H_{16}O_2$	E871329	99 %	Macklin
12	butyl dodecanoate	106-18-3	$C_{16}H_{32}O_2$	B835399	99 %	Macklin
13	ethyl pent-4-enoate	1968-40-7	$C_7H_{12}O_2$	E836291	98 %	Macklin
14	6-ethoxy-6-oxohexanoic acid	626-86-8	$C_8H_{14}O_4$	A800107	97 %	Macklin
15	dimethyl butanedioate	106-65-0	$C_6H_{10}O_4$	D822886	99.5 %	Macklin
16	diethyl butanedioate	123-25-1	$C_8H_{14}O_4$	D806708	99.0 %	Macklin
17	ethyl 6-oxohexanoate	27983-42-2	$C_8H_{14}O_3$	BD00756426	-	Fujifilm
18	ethyl 4-oxopentanoate	539-88-8	$C_7H_{12}O_3$	E809207	99 %	Macklin
19	5-heptyloxolan-2-one	104-67-6	$C_{11}H_{20}O_2$	U820339	98 %	Macklin
20	methyl heptanoate	106-73-0	$C_8H_{16}O_2$	M859147	99.0 %	Macklin
21	5-butyloxolan-2-one	104-50-7	$C_8H_{14}O_2$	O815306	99 %	Macklin
22	ethyl 2-methylpentanoate	39255-32-8	$C_8H_{16}O_2$	E808991	98 %	Macklin
23	octyl acetate	112-14-1	$C_{10}H_{20}O_2$	O815140	98%	Macklin
24	ethyl octanoate	106-32-1	$C_{10}H_{20}O_2$	E808629	99 %	Macklin
25	methyl 2-(oxan-4-yl)acetate	156002-64-1	$C_8H_{14}O_3$	M854828	95 %	Macklin
26	methyl undecanoate	1731-86-8	$C_{12}H_{24}O_2$	M875207	98 %	Macklin
27	prop-2-enyl heptanoate	142-19-8	$C_{10}H_{18}O_2$	A832229	98%	Macklin
28	methyl decanoate	110-42-9	$C_{11}H_{22}O_2$	M813292	98 %	Macklin
29	bis(2-ethylhexyl) hexanedioate	103-23-1	$C_{22}H_{42}O_4$	B802127	99%	Macklin
30	ethyl heptanoate	106-30-9	$C_9H_{18}O_2$	E808593	98.5 %	Macklin
31	diethyl decanedioate	110-40-7	$C_{14}H_{26}O_4$	D806720	98 %	Macklin
32	diethyl 4-oxoheptanedioate	6317-49-3	$C_{11}H_{18}O_5$	D837957	98%	Macklin
33	ethyl 2-methylbutanoate	7452-79-1	$C_7H_{14}O_2$	E809208	98 %	Macklin
34	dibutyl decanedioate	109-43-3	$C_{18}H_{34}O_4$	D807216	98 %	Macklin
35	ethyl (3S)-3-hydroxybutanoate	56816-01-4	$C_6H_{12}O_3$	E834909	98%	Macklin
36	3-methylbutyl 2-methylpropanoate	2050-01-3	$C_9H_{18}O_2$	I859350	99 %	Macklin
37	methyl octanoate	111-11-5	$C_9H_{18}O_2$	M824130	99 %	Macklin
38	dimethyl pentanedioate	1119-40-0	$C_7H_{12}O_4$	D806569	98 %	Macklin

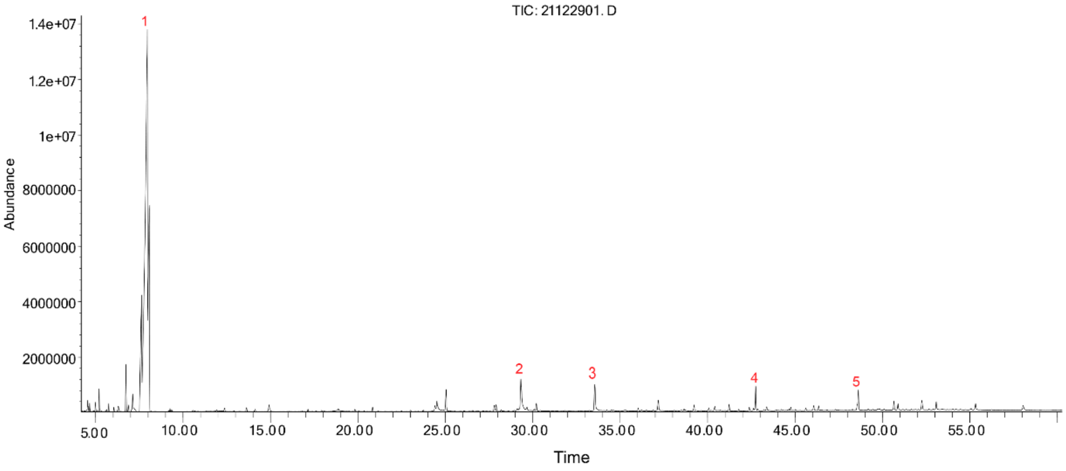

Figure S1 The GC-MS chromatogram of NB medium

1: cyclohexanol**; 4:** methyl (*Z*)-octadec-9-enoate; **5:** dioctyl benzene-1,2-dicarboxylate; **2**,**3:** not identified.

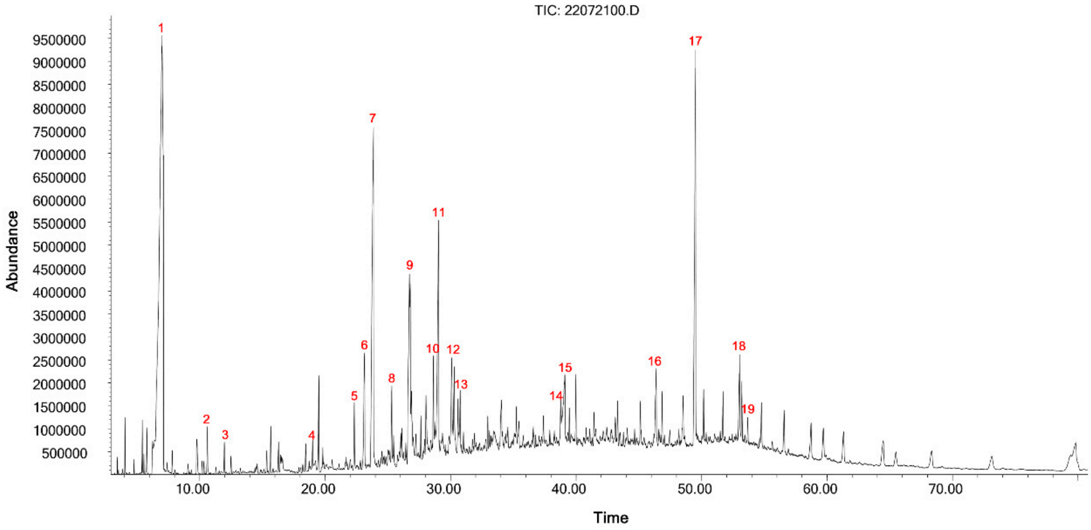

Figure S2 The GC-MS chromatogram of *B. diminuta*

1: cyclohexanol; **2:** butyl butanoate; **3:** butylcyclohexane; **4:** dodecane; **5:** cyclohexylcyclohexane; **6:** cyclohexyl pentanoate; **7:** cyclohexyloxycyclohexane; **8:** tetradecane; **10:** 2-(cyclohexen-1-yl)cyclohexan-1-one; **13:** 2-(1-hydroxycyclohexyl)cyclohexan-1-one; **14:** dibutyl benzene-1,2-dicarboxylate; **15:** hexadecanoic acid; **16:** octadec-9-enamide; **17:** bis(6-methylheptyl) benzene-1,2-dicarboxylate; **18:** (*Z*)-docos-13-enamide; **19:** (6*E*,10*E*,14*E*,18*E*)-2,6,10,15,19,23-hexamethyltetracosa-2,6,10,14,18,22-hexaene; **9, 11, 12：** not identified.
